# The complete chloroplast genome of *Ampelopsis grossedentata* (Hand.-Mazz.) W. T. Wang (Family: Vitaceae) and its phylogenetic analysis

**DOI:** 10.1080/23802359.2020.1775508

**Published:** 2020-06-11

**Authors:** Lei Gu, Ni Zhang, Chun Feng, Yin Yi, Zheng-Wen Yu

**Affiliations:** a School of Life Science, Guizhou Normal University, Guiyang, China; b State Key Laboratory of Functions and Applications of Medicinal Plants, Guizhou Medical University, Guiyang, China

**Keywords:** *Ampelopsis grossedentata*, Vitaceae, complete chloroplast genome, phylogenetic

## Abstract

*Ampelopsis grossedentata* (Hand.-Mazz.) W. T. Wang is rich in flavonoids and also displays excellent pharmacological activities. The phylogenetic relationship between *A. grossedentata* and other related Vitaceae family members remains unclear. The chloroplast (cp) genome is a useful model for assessing genome evolution. In this study, we assembled the cp genome of *A. grossedentata* using the high-throughput Illumina pair-end sequencing data and characterized the genome to providing useful information for future genetic studies. The circular cp genome was 162,147 bp in size, including a large single-copy (LSC) region of 89,244 bp and a small single-copy (SSC) region of 18,439 bp, which were separated by two inverted repeat (IR) regions (27,232 bp each). A total of 135 genes were predicted, including 8 ribosomal RNAs (rRNAs), 37 transfer RNAs (tRNAs), and 90 protein-coding genes (PCGs). Furthermore, phylogenetic analysis revealed that *A. grossedentata* within *Ampelopsis* genus and formed a different clade from other three congeneric species. This study provides useful information for future genetic study of *A. grossedentata*.

*Ampelopsis grossedentata* (Hand.-Mazz.) W. T. Wang, being classified into Vitaceae family, is widely growing in mountainous areas of southern China. The dried leaves and stems of *A. grossedentata,* also named vine tea, have extremely high health-promoting benefits due to its strong anti-oxidant (Gao et al. 2017; Ma et al. 2017), anti-inflammatory (Hou et al. 2015), and anti-tumor (Zhou et al. 2014) activities. Although *A. grossedentata* has received much attention, the complete chloroplast (cp) genome of *A. grossedentata* has not been reported. In this study, we assembled and determined the cp genome sequence of *A. grossedentata* as a resource for future genetic, genomic, breeding research.

Young and healthy leaf samples were collected from the *A. grossedentata* germplasm resource repository of Guizhou Normal University (26°22′51.06″N, 106°38′11.80″E, 1100.5 m above sea level) (Guiyang, Guizhou Province, China). The leaf specimen (accession number: GZNUYZW202001001) was deposited in the herbarium of School of Life Sciences, Guizhou Normal University. The total genomic DNA (No. YZW202001002) was extracted using DNAsecure Plant Kit (TIANGEN, Beijing) and stored at −80 °C in the laboratory (room number: 1403) of School of Life Science, Guizhou Normal University. A total amount of 700 ng DNA per sample was used as input material for the DNA sample preparations. Sequencing libraries were generated using NEB Next® Ultra DNA Library Prep Kit for Illumina® (NEB, USA) following manufacturer’s recommendations. The library preparations were sequenced on an Illumina platform and 150 bp paired-end reads were generated. After removal of adapter sequences, the filtered reads were assembled using the program GetOrganelle (Jin et al. 2019). The assembled cp genome was annotated using the online software GeSeq (https://chlorobox.mpimp-golm.mpg.de/geseq.html) (Tillich et al. 2017). The accurate annotated complete cp genome was submitted to GenBank with accession number MT267294.

The length of the complete cp genome sequence of *A. grossedentata* is 162,147 bp, consisting of a large single-copy (LSC, 89,244 bp) region, a small single-copy (SSC, 18,439 bp) region and two inverted repeat (IRA and IRB) regions of 27,232 bp each. Totally, 135 genes were predicted, including 90 protein coding genes (PCGs), 8 rRNA genes and 37 tRNA genes. Among of these assembled genes, 4 rRNAs (*rrn16*, *rrn23*, *rrn4.5* and *rrn5*), 7 PCGs (*rps7*, *rps19*, *rpl2*, *rpl23*, *ndhB*, *ycf2* and *ycf15*), and 7 tRNAs (*trnA-UGC*, *trnI-CAU*, *trnI-GAU*, *trnLCAA*, *trnN-GUU*, *trnR-ACG* and *trnV-GAC*) with double copies. 1 PCG (*rps12*) occur in three copies. Intron-exon analysis showed the majority (116 genes, 86%) genes with no introns, whereas 19 (14%) genes contain introns.

To further understand the chloroplast genome of *A. grossedentata*, 20 cp genome sequences of Vitaceae family (16 *Vitis* species, 3 species from *Ampelopsis* genus, and 1 specie from *Tetrastigma* genus) were downloaded from GenBank to construct the phylogenetic trees through maximum-likelihood (ML) analysis. The ML tree was performed using RAxML (Version 8.0.19, Model: GTRGAMMA) (Stamatakis 2014) with 1000 bootstrap replicates (confirm the stability of each tree node). The phylogenetic tree indicated that *A. grossedentata* belongs to *Ampelopsis* genus and located in a different clade from other three congeneric species (Figure 1). Compared to *Tetrastigma hemsleyanum*, a member of *Tetrastigma* genus, *A. grossedentata* was closely related to *Vitis* species (Figure 1).

**Figure 1.**
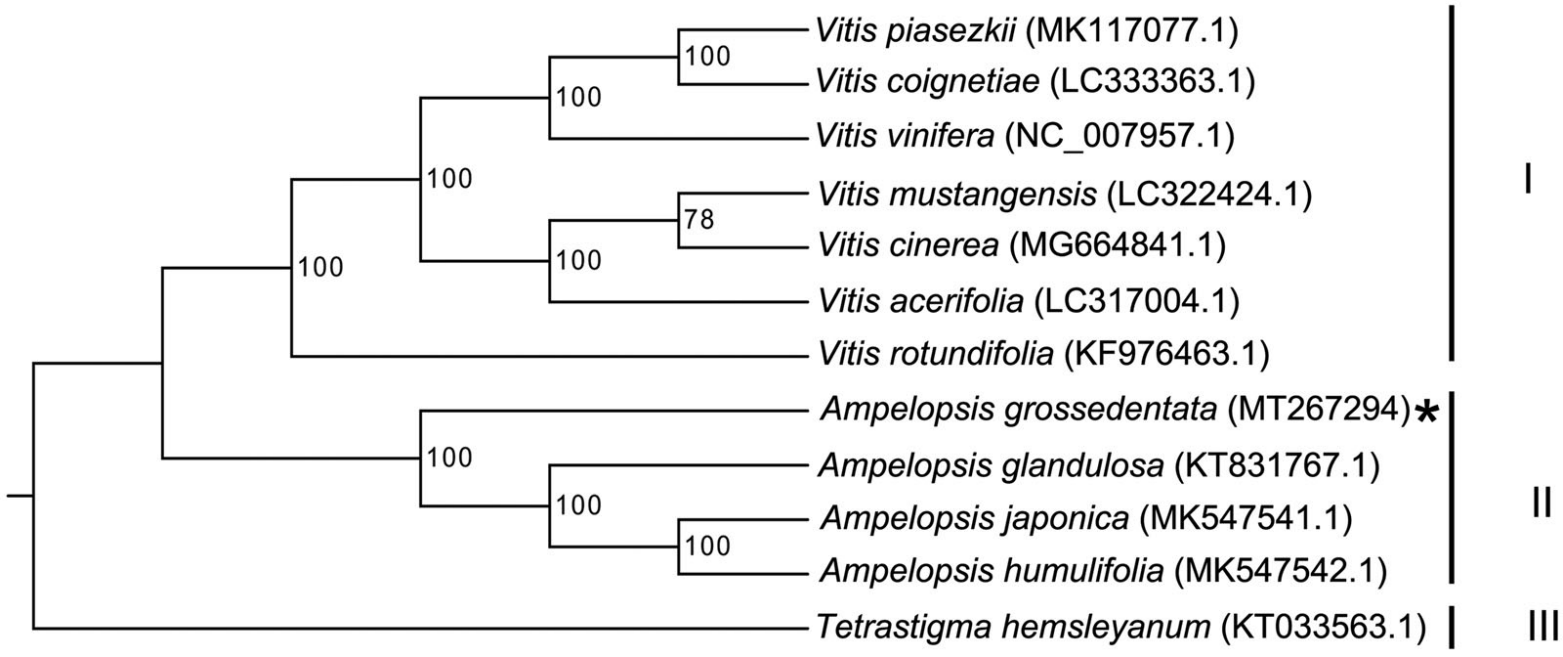
Maximum likelihood tree based on the complete cp genome sequences of 12 species from the Vitaceae family shows the three distinct clusters. GenBank accession numbers are described in the figure. Shown next to the nodes are bootstrap support values based on 1000 replicates.

## Data Availability

The data (the accurate annotated complete cp genome of *A. grossedentata*) that support the findings of this study are submitted to public database (NCBI GenBank, https://www.ncbi.nlm.nih.gov/) with GenBank accession number MT267294.
